# Self-Silencing, but Not Sexual Relationship Power Associated with Condom Use for Black College-Aged Women

**DOI:** 10.3390/bs9020013

**Published:** 2019-01-28

**Authors:** Lynissa R. Stokes, Leslie R. Brody

**Affiliations:** 1 Oregon Research Institute, Eugene, OR 97403, USA; 2 Department of Psychological & Brain Sciences, Boston University, Boston, MA 02215, USA; lrbrody@gmail.com

**Keywords:** HIV risk, condom use, sexual relationship power, self-silencing, Black college women

## Abstract

Black adolescent and young adult women in the United States experience a disproportionately higher rate of HIV infections than White and Hispanic adolescent and young adult women. Heterosexual sexual activity is the main route of infection for women, regardless of race or ethnicity. We examined two potential barriers to reducing Black adolescent and young adult women’s HIV risk: high levels of self-silencing and low levels of sexual relationship power. Data were collected on a small convenience sample of sexually active Black college-aged women (*N* = 57, *M*_age_ = 19.6, *SD* = 1.4) who answered questions about their current or most recent dating relationship. We found that higher levels of self-silencing were significantly related to lower condom use frequency and to a lower likelihood of reporting condom use at last sex. No significant associations were found between sexual relationship power and condom use (frequency or at last sex). Data from this study suggest that self-silencing, which involves putting the needs of others ahead of one’s own in order to avoid conflict in relationships, is an important variable to consider when examining potential risk factors for sexually transmitted HIV among Black college-aged women. Implications for future studies on HIV risk are reviewed.

## 1. Introduction

In 2016, over 60% of new HIV cases among adolescent and young women in the United States were Black/African American females [1]. Heterosexual sexual activity is the main route of HIV infection for females, regardless of race or ethnicity, between the ages of 13 and 24 [1]. Condoms remain the most widely available method to prevent the sexual transmission of HIV [2], yet low rates of condom use continue to be an issue for young men and women [1]. Although there is some evidence that female condoms can reduce HIV risk [3], the words “condom” and “condoms” used throughout this article refer only to male condoms.

A review of the myriad reasons why condoms are not used consistently or at all is beyond the scope of this paper. In the context of heterosexual relationships, two very relevant impediments to condom use for women are gender-based power imbalances and traditional gender roles [4,5,6]. Having less power in a sexual relationship and/or adhering to more traditional gender roles can make it difficult for women of any age to engage in protective actions against acquiring sexually transmitted HIV. Condom use negotiation may be particularly daunting for adolescent and young adult women because they may not yet have developed the skills necessary to successfully negotiate condom use with their partner.

### 1.1. Sexual Relationship Power

Sexual relationship power is defined as the ability of one partner to dominate decision-making, to engage in behaviors against the other partner’s wishes, and/or to control a partner’s actions [7] and has been studied extensively in relation to condom use [5,8,9,10]. Women with lower levels of sexual relationship power report less consistent condom use compared to women with sexual relationship power that is equal to or greater than their partner [5,6]. A number of these studies, however, have included older samples of women [11] or have not included Black adolescent or young adult women [6,9].

Among the studies that have sampled Black adolescent or young adult women, findings have not been consistent across studies. Some studies have found no significant associations between sexual relationship power and condom use [10,12,13]. Others, however, have found that when Black adolescent and young adult women feel that they have control over their partner’s condom use, condoms are used consistently [14], but that when partner resistance to condom use is encountered, condoms are used inconsistently or not at all [15,16].

### 1.2. Self-Silencing

Traditional gender roles that emphasize women’s passivity and submissiveness, in general, but especially in the context of sexual activity, are potential barriers to condom use [17,18]. This has been supported by studies that have examined women’s attitudes about gender roles [19,20] and women’s traditional versus non-traditional gender role traits [21,22]. In these studies, when women reported more egalitarian views about gender [19], exhibited less traditionally feminine traits (such as competitiveness) [22], or lower levels of hyperfemininity [21], they reported higher levels of condom use than women with less egalitarian views, more traditionally feminine traits, or higher levels of hyperfemininity.

Furthermore, women who reported more traditional attitudes about gender roles (e.g., that the husband should have more decision-making power in the marriage than the wife) perceived greater barriers to condom use [23]. Women who endorsed a passive acceptance of traditional gender roles (e.g., “I don’t see much point in questioning the general expectation that men should be masculine and women should be feminine”) were less insistent on engaging in safer sex practices than women who did not hold such views [20]. Nguyen and colleagues [22] found that women with high scores on a measure of interpersonal sensitivity, defined as a strong need for others’ approval, had lower levels of condom use compared to women with low scores on this measure of traditional gender role traits. Taken as a whole, these studies highlight the importance of including measures of traditional gender role beliefs and personality traits when studying condom use in heterosexual relationships.

One such measure, self-silencing, has not received extensive attention in studies on HIV risk, but it is very relevant to the study of condom use. Self-silencing is defined as the tendency to inhibit self-expression in order to avoid conflict with others and the possible loss of relationships [24]. The construct has been linked to unhealthy behaviors [25,26] and poorer health outcomes [27,28] in diverse populations of women and men.

The few studies that have examined self-silencing in relation to HIV risk or status suggest that higher levels of self-silencing could be a risk factor for acquiring HIV. For instance, Jacobs and Thomlison [29] found that lower levels of self-silencing were associated with engaging in safer sex behaviors among older women. In a study on self-silencing among women with and at risk for HIV, seropositive women had significantly higher levels of self-silencing compared to a control group of seronegative women [30]. To the best of our knowledge, no study of self-silencing and HIV risk or condom use has sampled Black adolescent or young adult women.

### 1.3. The Present Study

Research on HIV-related sexual risk taking among Black adolescent and young adult women usually focuses on non-college populations [11,12,15,19]. However, Black women attending college are a relevant population to study because they are not risk free [31,32,33,34]. For example, in a study of men and women attending a predominately minority serving university, over 75% of the African American women, compared to 50% of the White women, reported inconsistent condom use in the past 30 days [31].

For the present study, we were interested in examining whether sexual relationship power and self-silencing were significantly associated with condom use among Black college-aged women in dating relationships. Condom use was assessed both in terms of frequency and at last sex. We hypothesized that women with lower levels of self-silencing would report greater condom use frequency and be more likely to report condom use at last sex compared to women with higher levels of self-silencing. We also hypothesized that women with higher levels of sexual relationship power would report greater condom use frequency and be more likely to report condom use at last sex compared to women with lower levels of sexual relationship power.

## 2. Methods

### 2.1. Participants and Procedure

Individuals were eligible for this study if they identified as Black/African American, of African descent (e.g., Afro-Caribbean), or bi-racial (with one parent identifying as either Black/African American or of African descent), were fluent in English, and were between the ages of 18 and 22. Individuals did not have to identify as heterosexual to participate in the study, but they did need to be currently in or most recently in a dating relationship with a male partner. Participants were recruited via notices on college and university job boards, via flyers posted on the campus of the primary study site, through visits to undergraduate classes of one of the colleges near the primary study site, and through an announcement on a popular online advertising website. Participants who knew of other individuals who met eligibility requirements received an additional incentive of a $5 gift certificate if the person/people they recruited also completed the study. Less than 15% of the sample was recruited through this snowball sampling method.

Data were collected at several colleges and universities in two major cities in Massachusetts. The study received Institutional Review Board approval (Study #1266E) and written informed consent was obtained from each participant. Each participant received $12 in cash as compensation for answering the study questions.

### 2.2. Measures

*Demographic information*. Participants indicated their age, race, place of birth, marital status, level of education, primary language spoken at home, length of residence in the United States, and annual family household income.

*Dating Relationship Background*. Each participant was asked if she was currently dating someone, the duration of the relationship, and if she and her dating partner had agreed to date only each other. She was also asked if she believed that her partner was dating other people. If the participant was not in a dating relationship at the time of the study, she was asked to indicate how much time had passed since her last dating relationship and how long the relationship had lasted. Participants were also asked to indicate if during that recent dating relationship they had agreed to date only each other and if they believed that their partner was dating other people. Lastly, all participants, whether currently dating or recently dating, were asked if they were sexually active in that dating relationship.

#### 2.2.1. Predictor Variables

*Sexual Relationship Power* was assessed using the Sexual Relationship Power Scale-Modified (SRPS-M) [35]. The SRPS-M excludes three questions about condom use so that any associations found between sexual relationship power and condom use are not due to the condom-specific questions included in the original scale. Both the modified and original versions of the SRPS include two subscales: the relationship control subscale and the decision-making dominance subscale. Each item on the relationship control subscale is rated on a 4-point Likert scale, ranging from “1” (strongly agree) to “4” (strongly disagree). Items on the decision-making dominance scale are rated on a 3-point Likert scale, with “1” indicating that the partner makes the decision, “2” indicating that both the participant and the partner make the decision equally, and “3” indicating that the participant makes the decision.

A mean score is calculated for both of the subscales by adding the rating for each item together and then dividing the sum by the number of items answered. The mean score for the decision-making dominance subscale is then rescaled so that it has the same range as the relationship control subscale. The two subscale means are then added together and divided by two. Higher SRPS scores indicate higher sexual relationship power. Both the modified and original SRPS have been found to have good predictive validity and internal consistency [36] and have been used with diverse populations of women and men [10,37]. Alpha reliability for our study was 0.84.

*Self-Silencing* was measured using the Silencing the Self Scale (STSS) [38], a 31-item scale, with response options ranging from “1” (strongly disagree) to “5” (strongly agree). Respondents are asked to indicate their level of agreement with items such as “Caring means putting the other person’s needs in front of my own,” and “I don’t speak my feelings in an intimate relationship when I know they will cause disagreement.” Five items on the scale are reverse scored and then items are summed to create a total score, which can range from a low of 31 to a high of 155, with higher scores indicating higher levels of self-silencing. The measure has been shown to have good construct validity [38] and has been used with diverse populations of women and men [25,39,40]. Alpha reliability for our sample was 0.91.

#### 2.2.2. Outcome Variables

*Condom Use Frequency* was assessed by using an item from the Scale of Sexual Risk Taking (SSRT) [41], a 13-item self-report measure used to assess high-risk sexual behaviors of adolescents. Respondents are asked to indicate if their partner uses condoms “every time,” “most times,” “about half” of the time, “sometimes,” or “never.” A response of “1” indicates that condoms are used “every time” and a response of “5” indicates that condoms are “never” used. This scoring system was adopted by the scale developers so that a higher score on the SSRT would indicate higher levels of sexual risk taking. For the purposes of the present study, we chose to reverse score responses to this question so that “1” indicates that condoms are “never” used and “5” indicates that condoms are used “every time.” This was done so that it would be easier to interpret our results (e.g., a higher number represents greater condom frequency).

*Condom Use at Last Sex* was assessed by asking participants if condoms were used during their most recent sexual encounter with their dating partner, with “Yes” responses coded as “1” and “No” responses coded as “0.”

## 3. Data Analysis Plan

All analyses were conducted using SPSS version 24 (https://www.ibm.com/analytics/spss-statistics-software). Means and standard deviations were calculated for each continuous variable and percentages were calculated for each categorical variable. Analyses included Pearson correlations, chi-square tests, independent t-tests, and regression analyses. Dating relationship duration and perceived partner’s exclusivity, two questions from the Dating Relationship Background questionnaire, were covariates in the regression analyses as both have been found to be significantly associated with condom use in other studies [12,42,43].

## 4. Results

One hundred participants completed the study questionnaires. Of that number, 29 participants were excluded from further analyses because they reported that they had never been sexually active in any of their dating relationships. Of the remaining 71 participants, an additional 13 participants were excluded because they reported that they had not been sexually active in their current or most recent dating relationship. That left 58 participants who were either currently in a dating relationship in which they were sexually active (*N* = 44) or who had been sexually active in their most recent dating relationship (*N* = 14). One additional participant was eliminated from further analyses because she did not answer the question about the duration of her current or most recent dating relationship. Relationship duration was a question that was included in analyses as a covariate. Because there was too much variability in dating relationship duration among the other participants to confidently input the average relationship duration score for that participant, she was excluded from further analyses.

As shown in Table 1, the average age of participants was 19.6 (*SD* = 1.4). Most of the participants identified as Black/African American. Although there was some variability in reported racial background (e.g., 7% of the sample identified as Afro-Caribbean), participants will be referred to as Black throughout the paper. The majority of the participants were born in the United States, the largest percentage of the participants reported that their family income was between $25,000 and $49,999 per year, and the largest percentage of participants were first-year college students. None of the participants were married, all of the participants reported that English was the primary language spoken at home, and all of the participants had resided in the United States for three years or more (not shown in Table 1).

Most of the women (*N* = 44) indicated that they were currently in a dating relationship in which they were sexually active. The remaining participants (*N* = 13) answered study questions based on the most recent dating relationship in which they were sexually active. Before doing any further analyses, independent t-tests and chi-square tests were conducted to determine if there were any significant differences between the two groups of women among the demographic variables or the variables to be included in the regression analyses. As shown in Table 2, no significant differences were found between the two groups of women in terms of demographic variables, dating relationship duration, perceived partner exclusivity, predictor variables, or outcome variables. Therefore, all remaining analyses included the entire sample of 57 women.

Given the small sample size and the need to keep the variables included in the regression analyses to a minimum in order to maintain adequate power, we next examined whether any of the demographic variables included in Table 1 were correlated with either of the two outcomes: condom use frequency and condom use at last sex. As shown in Table 3, no significant correlations were found between any of the demographic variables and condom use (frequency and at last sex). Therefore, we did not include any of these variables in the regression analyses as covariates.

*Reported Condom Use*

A review of responses to the question “How often does your partner wear a condom?” from the SSRT indicated that 35.1% of the women (*N* = 20) reported that their dating partner wore condoms “every time.” The largest percentage of women (40.4%, *N* = 23) reported that their dating partner used condoms “most times,” 7% (*N* = 4) reported that their dating partner used condoms “sometimes,” and the remaining 17.5% (*N* = 10) stated that condoms were never used with their current or most recent dating partner. When asked about condom use at last sex, 64.9% (*N* = 37) of the women reported that condoms had been used.

*Predictors of Condom Use Frequency*

Hierarchical multiple regression analysis was used to determine if either sexual relationship power or self-silencing was significantly associated with condom use frequency. Relationship duration and perception of partner exclusivity were entered in Block 1 as covariates and the two predictor variables were entered in Block 2. The results indicated that lower levels of self-silencing were significantly associated with greater condom use frequency (β = −0.46, *t* = −2.84, *p* = 0.006). Sexual relationship power, however, was not significantly associated with condom use frequency (β = −0.26, *t* = −1.51, *p* = 0.14) (see Table 4 below for more details).

*Predictors of Condom Use at Last Sex*

Logistic regression analysis was performed to determine if either of the two predictor variables were significantly associated with condom use at last sex, with relationship duration and perception of partner exclusivity included as covariates. Higher STSS scores were associated with lower odds of reporting condom use at last sex (OR = 0.95, 95% CI = 0.91–1.00) (see Table 5). Specifically, for every one unit increase in STSS scores, the odds of reporting condom use at last sex was reduced by 5%. Sexual relationship power, however, was not significantly related to condom use at last sex. The final model (Block 2) accurately classified condom users and non-users at last sex 73.7% of the time compared to 64.9% of the time with only the constant in the model.

## 5. Discussion

Very few studies have examined self-silencing in relation to HIV risk [29,44]. This is the only study to do so with a sample of Black college-aged women. As hypothesized, we found that self-silencing was significantly related to condom use frequency and condom use at last sex. Specifically, lower levels of self-silencing were related to greater condom use frequency, while higher levels of self-silencing were associated with decreased odds of reporting condom use at last sex.

Self-silencing involves, among other perceived relationship maintenance behaviors, a tendency to agree with the wishes and needs of others over one’s own. Therefore, it makes sense that a woman who self-silences may also have difficulty using condoms consistently if she feels that advocating for condom use might jeopardize the continuation of the dating relationship. Our study did not include a measure of partner resistance to condom use, so it is unclear how, if at all, such resistance may be related to the levels of self-silencing reported by the women in our study.

That we found a significant relationship between self-silencing and condom use is congruent with findings from other studies that have examined self-silencing in the context of sexual relationships. Widman, Welsh, McNulty, and Little [45], in a study of adolescent dating couples, found that for the adolescent female participants, self-silencing was indirectly related to contraceptive use. Specifically, adolescent females with lower levels of self-silencing were more likely to engage in general sexual communication with their partner, which in turn was related to a greater likelihood of using contraception. From the wording of the question used to assess contraceptive use (“When the two of you have sexual intercourse, how often do you or your current partner use some form of contraception?”), it is unclear if respondents who reported such use were referring to condoms, some other method(s) of contraception, or if they used condoms in combination with other contraceptive methods. In addition, the researchers did not explore direct associations between self-silencing and contraceptive use.

In another relevant study, Jacobs and colleagues [46] found that middle-aged men who have sex with men (MSM) reported higher levels of self-silencing and higher levels of unprotected anal sex (UAS) than older MSM. Self-silencing, however, was not included in the analyses to predict UAS. Therefore, any relationship between self-silencing and UAS for this population is speculative. In the Jacobs and Thomlison [29] study described in the Introduction, self-silencing was examined in relation to safer sex practices in a sample of middle-aged and older women. Safer sex practices, however, were conceptualized and measured as a combination of protective behaviors (e.g., condom use), the avoidance of potentially risky behaviors (e.g., anal sex), and interpersonal and negotiation skills with a sexual partner. Our study, therefore, is the first to find a direct association between self-silencing and condom use. Along with the results from our study, the findings from Widman and colleagues [45] and Jacobs and colleagues [29,46] suggest that self-silencing is a potential obstacle to reducing HIV risk for diverse populations of men and women across the lifespan.

Our study joins a number of others that have found no significant relationship between relationship power and condom use. In the Bralock and Koniak-Griffin study [12], behavioral intentions, partner’s age, and the duration of the sexual relationship were significantly associated with condom use. Gutiérrez and colleagues [13] found that none of the power-related variables included in their analyses (interpersonal, personal, and relationship power) was significantly related to condom use with a steady partner for any of the participants in their study. Individual variables such as age at first intercourse and self-efficacy for condom use were significantly related to condom use for the African American adolescent females included in the study. Intimate partner violence, but not sexual relationship power, was significantly associated with a greater likelihood of inconsistent condom use for the African American and Hispanic female adolescents in the study done by Teitelman and colleagues [10]. Harvey, Thorburn Bird, Galavotti, Duncan, and Greenberg [47] found that sexual decision-making dominance, but not relationship power, was associated with condom use in a sample of racially/ethnically diverse 18- to 25-year-old women, many of whom were cohabitating with their sexual partner.

With the exception of the study done by Teitelman and colleagues [10], the other studies reviewed in the previous paragraph had larger sample sizes than ours. This suggests that the nonsignificant association we found between sexual relationship power and condom use may not be due to sample size issues alone. Rather, it could be that sexual relationship power, or relationship power in general, while very relevant to understanding impediments to condom use in general, may not be the predominant factor to consider when examining whether condoms are used in dating relationships. Other than the Bralock and Koniak-Griffin study [12], in the other studies previously described that found no significant association between relationship power and condom use, the participants were in dating relationships or cohabitating with their sexual partner.

There is some evidence that women in more committed relationships are more likely to use contraceptive methods other than condoms (e.g., the pill or vaginal ring) compared to women in casual relationships [48]. Therefore, one possible explanation for the nonsignificant finding between sexual relationship power and condom use in our study is that perhaps the participants were more likely to use other forms of contraception. The SSRT used to assess condom use frequency in our study also includes a question about birth control use. However, the way in which the question is phrased makes it difficult to determine if birth control is used instead of condoms or in addition to condoms. Future studies on self-silencing and HIV risk should include questions about contraceptive use that are better able to determine if participants use some form of contraception at all times, are dual method users (e.g., condoms and another form of birth control), or base their contraceptive use decisions on the type of sexual activity they engage in (e.g., vaginal versus anal sex) to get a better sense of potential HIV risk among Black college-aged women.

Some additional limitations should be noted. First, our findings are based on a small, convenience sample of Black older adolescent and young adult women attending college or post college degree (e.g., in graduate school), mostly from two urban cities in the Northeast. Therefore, our results have limited generalizability to other populations of sexually active Black college-aged women or to non-college Black women between the ages of 18 and 22. A larger sample size, with more geographically diverse participants, would aid in making future studies, should they find similar results, more generalizable to both college and non-college Black young adult women throughout the United States.

In addition, although the focus of this study was on Black college-aged women, future studies should include a racially/ethnically diverse sample of college-aged women to see if the significant relationship we found between self-silencing and condom use in the present study is also found for college-aged women of different races and ethnicities. If similar results are found for college-aged women, regardless of race/ethnicity, such findings could have implications for college-based sexual health programming designed to increase rates of condom use. Future studies may also wish to include other predictor variables, such as partner’s age and intimate partner violence, which were included in the Bralock and Koniak-Griffin [12] and the Teitelman et al. [10] studies, respectively, and found to be significantly related to condom use patterns among the women in their studies. 

Another limitation is that this study was cross-sectional in design. Therefore, relationships between the variables cannot be interpreted as causal. It may be that high levels of self-silencing made it difficult for women to broach the subject of condom use with their partner. Conversely, it could also be that women who encounter partner resistance to condom use are less likely to bring up the subject in the future for fear of potentially jeopardizing the continuation of the relationship. The finding by Widman and colleagues [45] that higher levels of self-silencing were associated with a lower likelihood of engaging in general sexual communication with a partner lends support for the first possibility. Replication of the study using a longitudinal design would better allow for causal interpretations.

Lastly, no questions were asked about HIV testing history or HIV status. Therefore, it is difficult to determine if the women who indicated that they did not always use condoms (64.9% of the sample) were making an informed decision based on knowledge about their own and their partner’s risk profile or not. Future studies on the relationship between self-silencing and HIV risk should include questions about HIV testing history and HIV status in order to better determine how much potential risk is involved when inconsistent condom use is reported.

Despite these limitations, our study adds to the body of research on potential HIV risk among Black college-aged women by finding a direct association between self-silencing and condom use. Our findings support the relevance of including self-silencing as a measure in future studies on condom use patterns of Black college-aged women.

## Figures and Tables

**Table 1:** Demographic Characteristics of the Sample (*N* = 57).

Variable	Mean/SD or %
Age (years)	19.6/1.4
Race/Ethnicity	
Black/African American	82.5%
Afro-Caribbean	7.0%
Bi-racial	7.0%
African	3.5%
Place of Birth ^a^	
United States	84.2%
Caribbean	8.8%
Africa	5.3%
Europe	1.8%
Annual Family Income ^b^	
Less than $25,000	14.0%
$25,000 to $49,999	45.6%
$50,000 to $75,000	17.5%
More than $75,000	21.1%
College Status ^a^	
Freshman	29.8%
Sophomore	15.8%
Junior	21.1%
Senior	21.1%
College graduate	1.8%
Other	10.5%

**Note**. “Other” in the College Status category represents those that had been accepted to, but had not yet started, college or those accepted to or already attending graduate school. ^a^ Total greater than 100% due to rounding error. ^b^ One participant did not answer this question, therefore the total does not add up to 100%.

**Table 2:** Comparisons between Participants Currently in a Dating Relationship and Participants Reporting on Their Most Recent Dating Relationship (*N* = 57).

Variable	Currently Dating (*N* = 44)	Recently Dating (*N* = 13)	
	Mean/SD or *N* (%)	Mean/SD or *N* (%)	*p*-Value ^a^
Age (years)	19.8/1.4	19.0/1.4	0.09
Race/Ethnicity			0.43
Black/African American	36 (81.8%)	11 (84.6%)	
African	1 (2.3%)	1 (7.7%)	
Afro-Caribbean	4 (9.1%)	0 (0%)	
Bi-racial	3 (6.8%)	1 (7.7%)	
Place of Birth			0.33
United States	36 (81.8%)	12 (92.3%)	
Caribbean	5 (11.4%)	0 (0%)	
Africa	2 (4.5%)	1 (7.7%)	
Europe	1 (2.3%)	0 (0%)	
Annual Family Income ^b^			0.84
Less than $25,000	7 (15.9%)	1 (7.7%)	
$25,000 to $49,999	20 (45.5%)	6 (46.2%)	
$50,000 to $75,000	7 (15.9%)	3 (23.1%)	
More than $75,000	9 (20.5%)	3 (23.1%)	
College Status ^c^			0.68
Freshman	11 (25%)	6 (46.2%)	
Sophomore	8 (18.2%)	1 (7.7%)	
Junior	9 (20.5%)	3 (23.1%)	
Senior	10 (22.7%)	2 (15.4%)	
College Graduate	1 (2.3%)	0 (0%)	
Other	5 (11.4%)	1 (7.7%)	
Relationship Duration ^d^	2.8/1.5	2.1/1.4	0.11
Perceived Partner Exclusivity ^e^	4.4/1.2	3.8/1.6	0.13
SRPS-M Mean	2.9/.44	2.8/.35	0.37
STSS Score	65.6/19.9	69.8/12.9	0.37
Condom Use Frequency ^f^	3.5/1.5	4.2/1.3	0.19
Condom Use Last Sex (yes)	28 (63.6%)	9 (69.2%)	0.99

**Note**. “Other” in the College Status category indicates those that had been accepted to, but had not yet started, college or those accepted to or already attending graduate school. SRPS-M = Sexual Relationship Power Scale - Modified. STSS = Silencing the Self Scale. ^a^ The p-values reported are based on t-tests for the continuous variables and chi-square tests for the categorical variables. ^b^ One participant (who was currently dating) did not answer this question, therefore the total does not add up to 100%. Total greater than 100% due to rounding error for those recently dating. ^c^ Totals in both columns greater than 100% due to rounding error. ^d^ Response options: 1 = Less than 6 months; 2 = 6 months to less than 1 year; 3 = 1 year to less than 2 years; 4 = 2 years to less than 3 years; 5 = 3 or more years. ^e^ Response options: 1 = no; 2 = probably not; 3 = unsure; 4 = probably yes; 5 = definitely yes. ^f^ Response options: 1 = never; 2 = sometimes; 3 = about half; 4 = most times; 5 = every time.

**Table 3:** Correlations between Demographic Variables and Condom Use (Frequency and at Last Sex).

Variable	1	2	3	4	5	6	7
1. Participant Age							
2. Race	0.12						
3. Place of Birth	0.07	0.16					
4. Length of Time in the US	−0.11	−0.24	−0.47 **				
5. Year in College	0.66 **	0.26*	0.08	−0.19			
6. Annual Family Income	−0.06	−0.11	−0.20	0.12	−0.10		
7. Condom Use Frequency	0.02	−0.10	−0.09	−0.06	−0.04	0.03	
8. Condom Use at Last Sex	−0.18	−0.12	−0.12	0.03	−0.07	0.13	0.73 **

**Note**. * *p* ≤ 0.05; ** *p* ≤ 0.01.

**Table 4:** Hierarchical Regression Analysis for Variables Predicting Condom Use Frequency.

Variable	*B*	*SE B*	β	*t*	*p*-Value
Block 1					
Relationship Duration	−0.19	0.13	−0.19	−1.41	0.16
Perceived Partner Exclusivity	−0.15	0.15	−0.14	−1.04	0.30
Block 2					
Relationship Duration	−0.21	0.13	−0.21	−1.67	0.10
Perceived Partner Exclusivity	−0.13	0.15	−0.12	−0.88	0.38
SRPS-M Mean	−0.90	0.60	−0.26	−1.51	0.14
STSS Score	−0.04	0.01	−0.46	−2.84	0.006

**Note**. *B* = Unstandardized regression coefficient. *SE B* = Standard error. β = Standardized regression coefficient. SRPS-M = Sexual Relationship Power Scale-Modified. STSS = Silencing the Self Scale. Block 1: *R*^2^ = 0.06; ∆ *R*^2^ = 0.06; Block 2: *R*^2^ = 0.19; ∆ *R*^2^ = 0.13.

**Table 5:** Logistic Regression Analysis for Variables Predicting Condom Use at Last Sex.

Variable	Odds Ratio	95% CI	*p*-Value
Block 1			
Relationship Duration	0.69	0.47–1.03	0.07
Perceived Partner Exclusivity	0.87	0.54–1.38	0.54
Block 2			
Relationship Duration	0.67	0.43–1.01	0.06
Perceived Partner Exclusivity	0.81	0.46–1.41	0.45
SRPS-M Mean	0.50	0.06–4.28	0.53
STSS Score	0.95	0.91–1.00	0.03

**Note**. CI = Confidence interval. SRPS-M = Sexual Relationship Power Scale-Modified. STSS = Silencing the Self Scale.
